# Lack of rewarding effects of a soluble epoxide hydrolase inhibitor TPPU in mice: Comparison with morphine

**DOI:** 10.1002/npr2.12136

**Published:** 2020-09-07

**Authors:** Xiayun Wan, Yuko Fujita, Lijia Chang, Yan Wei, Li Ma, Gerile Wuyun, Yaoyu Pu, Bruce D. Hammock, Kenji Hashimoto

**Affiliations:** ^1^ Division of Clinical Neuroscience Chiba University Center for Forensic Mental Health Chiba Japan; ^2^ Department of Entomology and Nematology, and UCD Comprehensive Cancer Center University of California Davis CA USA

**Keywords:** conditioned place preference, dependence, morphine, reward

## Abstract

**Aim:**

Although opioids have been used as treatment of neuropathic pain, opioids have abuse potential in humans. Since soluble epoxide hydrolase (sEH) in the metabolism of polyunsaturated fatty acids plays a key role in the pain, sEH inhibitors would be promising new therapeutic drugs for neuropathic pain. In this study, we examined the effect of the sEH inhibitor TPPU on rewarding effects in mice using the conditioned place preference (CPP) paradigm.

**Methods:**

The rewarding effects of morphine (10 mg/kg) and TPPU (3, 10, or 30 mg/kg) in mice were examined using CPP paradigm. Furthermore, the effect of TPPU (30 mg/kg) on morphine‐induced rewarding effects was examined.

**Results:**

TPPU (3, 10, or 30 mg/kg) did not increase CPP scores in the mice whereas morphine significantly increased CPP scores in the mice. Furthermore, pretreatment with TPPU did not block the rewarding effects of morphine in the mice, suggesting that sEH does not play a role in the rewarding effect of morphine.

**Conclusion:**

This study suggests that TPPU did not have rewarding effects in rodents. This would make sEH inhibitors potential therapeutic drugs without abuse potential for neuropathic pain.

## Abstract 2

The sEH inhibitor TPPU did not increase CPP score in mice. The sEH inhibitors might not produce rewarding effects in humans.

## Graphical abstract

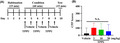

## 1 INTRODUCTION

Neuropathic pain is a pain condition caused by chronic, progressive nerve disease. Approximately 30% of people in United State (US) experience chronic pain, and about 20% of people with chronic pain experience neuropathic pain. A recent meta‐analysis showed a strong recommendation for use and proposal as first‐line treatment in neuropathic pain for tricyclic antidepressants, serotonin‐noradrenaline reuptake inhibitors, pregabalin, and gabapentin. Furthermore, a weak recommendation second line is lidocaine patches, capsaicin high‐concentration patches, and tramadol. Moreover, a weak recommendation as third line is the strong opioids.^1^ Although the opioids have been widely used in chronic neuropathic pain, opioids are not recommended because of abuse liability. The misuse and addiction to the opioids including prescription pain relievers, heroin, and synthetic opioids, is a serious concern that can affect public health in the US.^2^, ^3^ Therefore, the development of novel alternative safe compounds for opioids is unmet medical need for neuropathic pain.

Epoxy fatty acids in the metabolism of polyunsaturated fatty acids (PUFAs) are recognized as important cell signaling molecules with multiple biological actions including antinociception.^4^ Epoxy fatty acids are metabolized to the corresponding diols by soluble epoxide hydrolase (sEH).^5^, ^6^ Wagner et al^7^ demonstrated that the sEH inhibitors (APAU, *t*‐TUCB, and *t*‐AUCB) were superior to the COX‐2 inhibitor celecoxib in both diabetic neuropathic pain and lipopolysaccharide (LPS)‐induced inflammatory pain models. Furthermore, it is demonstrated that the sEH inhibitor TPPU [1‐trifluoromethoxyphenyl‐3‐(1‐propionylpiperidine‐4‐yl)urea]^8^ has beneficial effects in several preclinical models of chronic neuropathic pain.^4^, ^9^, ^10^ Although the tolerance to opioids as analgesics is well known, there are no reports showing comparison of sEH inhibitors and opioids such as morphine in rodents.

Conditioned place preference (CPP) paradigm has been widely used as rewarding effects of certain compounds.^11^, ^12^ Previously, we published that the potent sEH inhibitor TPPU showed beneficial effects in several animal models such as depression, Parkinson's disease, schizophrenia, and autism.^13^, ^14^, ^15^, ^16^ In this study, we examined the effect of TPPU on rewarding effects in mice using CPP paradigm. We also examined the effects of morphine in this paradigm, since morphine is known to have potent rewarding effects in mice.

## 2 METHODS AND MATERIALS

### 2.1 Animals

Male C57BL/6 mice (aged 8 weeks, body weighing 20‐25 g) were purchased from Japan SLC (Hamamatsu). Mice were housed in clear polycarbonate cages (22.5 × 33.8 × 14.0 cm) in groups of 5 or 6 per cage, under controlled conditions for temperature (23 ± 1°C) and humidity (55 ± 5%) with a 12‐hour light/dark cycle (lights on from 7:00 to 19:00). Mice were allowed free access to food (CE‐2; CLEA Japan, Inc) and water. The experiment using mice was approved by the Animal Care and Use Committee of Chiba University (permission number:1‐467 and 2‐157).

### 2.2 Materials

Morphine hydrochloride hydrate was purchase from Daiichi‐Sankyo Ltd. TPPU was synthesized at Hammock laboratory as previously reported.^8^ Other reagents were purchased commercially.

### 2.3 Conditioned place preference (CPP) and treatment

**FIGURE 1:**
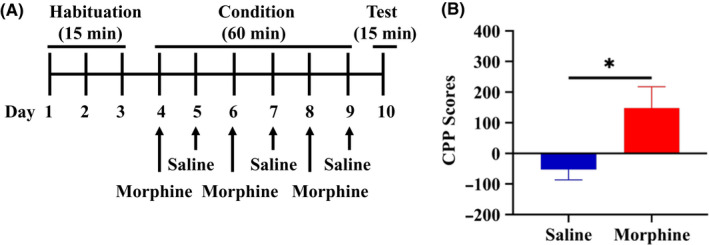
Experiment schedule for the CPP paradigm. A, Schedule of CPP paradigm. Habituation of mice for 15 min/day was performed 3 d from day 1 to day 3. Mice were treated with saline (10 mL/kg, i.p.) or morphine (10 mg/kg, i.p.) from day 4 to day 9. Test for 15 min was performed on day 10. Detailed procedure was shown in the Method section. B, The CPP scores in the morphine‐treated group were significantly (*t* = −2.588, *df* = 14.30, *P* = .021) higher than those of saline‐treated group. *P < .05 (unpaired two‐tailed Student's *t* test). The values are the mean ± SEM (n = 11)

**FIGURE 2:**
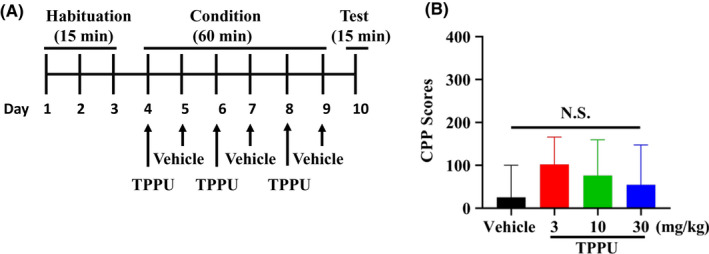
Lack of rewarding effects of TPPU in mice. A: Schedule of CPP paradigm. Habituation of mice for 15 min/day was performed 3 d from day 1 to day 3. Mice were treated with vehicle (10 mL/kg, p.o.) or TPPU (3, 10, or 30 mg/kg, p.o.) from day 4 to day 9. Test for 15 min was performed on day 10. Detailed procedure was shown in the Method section. B, There were no differences (one‐way ANOVA: F_3,39_ = 0.160, *P* = .922) among the four groups. The values are the mean ± SEM (n = 10‐12). NS: not significant

**FIGURE 3:**
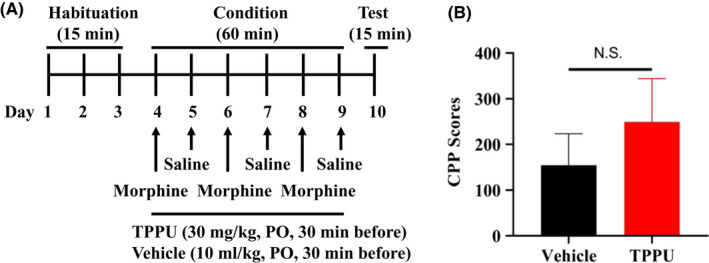
The effect of TPPU on morphine‐induced rewarding effects. A: Schedule of CPP paradigm. Habituation of mice for 15 min/day was performed 3 d from day 1 to day 3. Mice were treated with vehicle (10 mL/kg, p.o., 30 min before) + morphine (10 mg/kg, i.p.) or TPPU (30 mg/kg, p.o., 30 min before) + morphine (10 mg/kg, i.p.) from day 4 to day 9. Test for 15 min was performed on day 10. Detailed procedure was shown in the Method section. B, There was no differences (Mann‐Whitney *U* test: *P* = .143) between the two groups. The values are the mean ± SEM (n = 10). NS, not significant

The conditioned place preference (CPP) was performed using the place conditioning paradigm (Brain Science Idea Inc, Osaka, Japan) as reported previously.^17^, ^18^, ^19^ Experiment‐1: The groups were control (saline [10 ml/kg]) group and morphine (10 mg/kg) group. The test mouse was allowed to move freely between transparent and black boxes for a 15 minutes session once a day, for 3 days (days 1‐3) as preconditioning (Figure 1A). On day 3, the time spent in each box was measured. There was no significant difference between time spent in the black compartment with a smooth floor and the white compartment with a textured floor, indicating that there was no place preference before conditioning. On days 4, 6, and 8, morphine (10 mg/kg as hydrochloride hydrate, Daiichi‐Sankyo Ltd., Tokyo, Japan) was injected intraperitoneally (i.p.), and, then mice were confined to either the transparent or black box for 60 minutes (Figure 1A). On days 5, 7, and 9, mice were given saline (10 mL/kg) and placed in the opposite morphine‐conditioning box for 60 minutes. On day 10, the postconditioning test was performed without drug treatment, and the time spent in each box was measured for 15 minutes (Figure 1A). A counterbalanced protocol was used in order to nullify any initial preference by the mouse. The CPP score was determined as the time spent in the drug‐conditioning sites, minus the time spent in the saline‐conditioning sites. Experiment‐2: The groups were control (vehicle [10 ml/kg; 100% polyethylene glycol 400 [PEG400]) group, and TPPU (3, 10, or 30 mg/kg) group (Figure 2A). On day 10, the CPP score was determined as described above. Experiment‐3: The two groups were vehicle (PEG400, 10 ml/kg, p.o.) + morphine (10 mg/kg, i.p.) group and TPPU (30 mg/kg, p.o.) + morphine (10 mg/kg, i.p.) group (Figure 3A). On day 10, the CPP score was determined as described above.

### 2.4 Data analysis

Data are presented as the mean ± standard error of the mean (SEM). CPP data were analyzed by unpaired two‐tailed Student's *t* test, nonparametric Mann‐Whitney *U* test, or one‐way analysis of variance (ANOVA), followed by the *post hoc* Dunnett test. Significance was set at *P* < .05.

## 3 RESULTS

First, we investigated the rewarding effects of morphine using the CPP paradigm, which measures the rewarding properties of abused drugs. Repeated treatment of morphine (10 mg/kg) significantly increased CPP scores compared with saline‐treated group, indicating the rewarding effect of morphine (Figure 1B). Second, we investigated whether TPPU can increase CPP scores in mice. Repeated treatment with TPPU (3, 10, or 30 mg/kg, p.o.) did not increase CPP scores compared with vehicle‐treated group (Figure 2B). The data suggest that TPPU does not have rewarding effects in mice. Finally, we examined whether TPPU could block morphine‐induced rewarding effects in mice. When mice were treated with TPPU (30 mg/kg, p.o.) 30 minutes before receiving morphine (10 mg/kg, i.p.), TPPU did not alter morphine‐induced CPP increase in the mice (Figure 3B). The data suggest that sEH may not play a role in the development of rewarding effects of morphine in mice.

## 4 DISCUSSION

In this study, we found that TPPU did not increase CPP scores in the mice although morphine significantly increased CPP scores in the mice. Furthermore, the pretreatment with TPPU did not block the morphine‐induced rewarding effects in the mice, suggesting that sEH does not play a role in the rewarding effects of opioids such as morphine. Therefore, it is unlikely that sEH inhibitors might have rewarding effects of opioids in humans.

Oxidative metabolism by cytochrome P450 (CYP450) in the PUFAs such as ω‐3 docosahexaenoic acid (DHA) and eicosapentaenoic acid (EPA) as well as the ω‐6 arachidonic acid is known to produce different classes of epoxy fatty acids.^5^, ^6^ These epoxy fatty acids are known to play a role in the pain although these fatty acids are metabolized by sEH. Importantly, the analgesic effects of epoxy fatty acids are different from opioids.^4^ TPPU is reported to produce therapeutic effects in several preclinical models of neuropathic pain. In the CPP paradigm, TPPU (10 mg/kg) did not increase CPP score in control naïve mice,^10^ consistent with our current data. A recent study demonstrated that TPPU mediated effective analgesia without tolerance in rats.^20^ In this study, we found that TPPU did not have morphine‐like rewarding effects in mice. Collectively, it is likely that sEH inhibitors would be promising candidates without abuse potential for neuropathic pain in humans. In 2020, US Food and Drug Administration has granted Fast Track designation to EC5026 which is a first‐in‐class orally administered, potent sEH inhibitor.^21^ It is of great interest to investigate whether EC5026 could produce beneficial effects in patients with neuropathic pain. Furthermore, analgesic effects of the novel sEH/PDE4 (phosphodiesterase 4) dual inhibitor MPPA [*N*‐(4‐methoxy‐2‐(trifluoromethyl)benzyl)‐1‐propionylpiperazine‐4‐carboxamide] has been reported.^22^ In addition, MPPA did not alter self‐motivated exploration of rats with inflammatory pain or the withdrawal latency in control rats.^22^ Therefore, it is also interesting to investigate the effects of sEH/PDE4 dual inhibitor in patients with neuropathic pain.

Opioids are known to cause several detrimental side effects such as respiratory depression, severe constipation, and addiction. Importantly, opioid abuse is a most serious public health in the US.^2^, ^3^ It is suggested that sEH inhibitors have potential to be a multimodal, disease‐modifying approach to treat neuropathic pain.^23^ Given the opioid crisis in the US, it is likely that sEH inhibitors would be promising alternative candidates for opioids in humans.

In conclusion, this study suggests that the sEH inhibitor TPPU did not have morphine‐like rewarding effects in mice. Therefore, sEH inhibitors would appear to be a safe drug for neuropathic pain, although further studies of sEH inhibitors in humans will be necessary.

## CONFLICT OF INTEREST

Dr Hashimoto declares that he has received research support and consultant from Dainippon Sumitomo, Otsuka, and Taisho. The other author declares no conflict of interest.

## AUTHOR CONTRIBUTION

KH is responsible for the design of the research and experiment and supervised the experimental analyses. XW and KH wrote the paper. XW, YF, LC, YW, LM, GW, and YP performed the experiments. BDH provided the compound TPPU. XW analyzed the data. All authors read and approved this paper.

## ANIMAL STUDIES

All animal experiments were approved by the Animal Care and Use Committee of Chiba University.

## Supporting information

Tab S1

## Data Availability

The data that support the findings of this study are available in the Table S1 of this article.
